# MHCII restriction demonstrates B cells have very limited capacity to activate tumour-specific CD4^+^ T cells in vivo

**DOI:** 10.1080/2162402X.2023.2290799

**Published:** 2023-12-10

**Authors:** Thomas V. Guy, Alexandra M. Terry, Helen M. McGuire, Elena Shklovskaya, Barbara Fazekas de St Groth

**Affiliations:** a T cell Biology Program, Centenary Institute of Cancer Medicine and Cell Biology, Sydney, NSW, Australia; b Pillai Labratory, Ragon Institute of MGH, Harvard and MIT, Boston, MA, USA; c Genmab, Utrecht, The Netherlands; d School of Medical Sciences, Faculty of Medicine and Health, The University of Sydney, Sydney, NSW, Australia; e Faculty of Medicine, Health and Human Sciences, Macquarie University, Sydney, NSW, Australia

**Keywords:** Antigen presentation, B cell, MHC restriction, T cell, tumour

## Abstract

There has been growing interest in the role of B cells in antitumour immunity and potential use in adoptive cellular therapies. To date, the success of such therapies is limited. The intrinsic capacity of B cells to specifically activate tumour-specific CD4+ T cells *in* *vivo* via TCR-dependent interactions remains poorly defined. We have developed an *in* *vivo* tumour model that utilizes MHCII I-E restriction which limits antigen presentation to tumour-specific CD4 T cells to either tumour-specific B cells or host myeloid antigen presenting cells (APCs) in lymphopenic RAG-/-mice. We have previously shown that these naive tumour-specific CD4+ T cells can successfully eradicate established tumours in this model when activated by host APCs. When naïve tumour-specific B cells are the only source of I-E+ APC, very limited proliferation of naïve CD4+ T cells is observed, whereas host I-E+ APCs are potent T cell activators. B cells pre-activated with an anti-CD40 agonistic antibody *in* *vivo* support increased T cell proliferation, although far less than host APCs. CD4+ T cells that have already differentiated to an effector/central memory phenotype proliferate more readily in response to naïve B cells, although still 100-fold less than in response to host APCs. This study demonstrates that even in a significantly lymphopenic environment, myeloid APCs are the dominant primary activators of tumour-specific T cells, in contrast to the very limited capacity of tumour-specific B cells. This suggests that future anti-tumour therapies that incorporate activated B cells should also include mechanisms that activate host APCs.

## GRAPHICAL ABSTRACT

**Figure:**
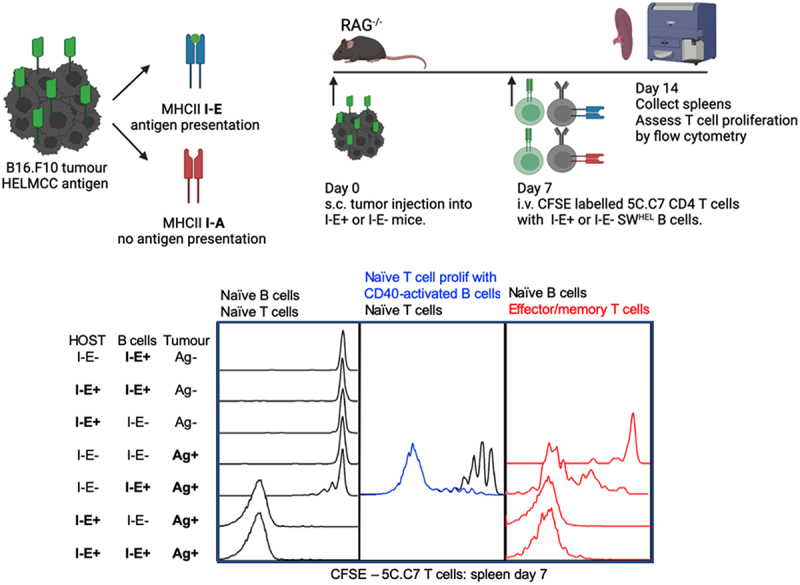

## Introduction

Despite the success of immune checkpoint therapy, there is an ongoing need to broaden response rates in patients with cancer. While antigen presenting cell (APC)-T cell interactions are key, the roles of professional APCs are very difficult to assess in patients and the relative contributions of dendritic cells (DCs) versus B cells are not well understood. Tumour infiltrating B cells have been observed in a growing number of cancer types and likely interact with T cells in both secondary lymphoid tissues and tumour tissue.^1^ Recent interest has focussed on B cells as a candidate for CD4^+^ T cell-based cellular vaccine approaches.

Both DCs and B cells express high levels of surface MHC class II (MHCII). MHCII expression by DCs is generally accepted as the initial driver of CD4^+^ T cell activation, while B cell MHCII is required for receipt of antigen-specific MHCII-restricted CD4^+^ T cell help. However B cell MHCII expression may also play a role in primary CD4^+^ T cell activation in certain settings. *In vitro* CD40L-activated B cells have shown some promise in stimulating anti-tumour CD4^+^ and CD8^+^ T cell responses leading to reduced tumour growth in mice.^2^ The specific mechanisms involved remain unclear. It is not known whether such tumour responses involve transfer of tumour antigen to endogenous APCs, nor whether they are driven by CD4^+^ T cells alone or a combination of CD8^+^ and CD4^+^ T cells. Moreover, the *in vivo* capacity of tumour-specific B cells to activate tumour-specific CD4^+^ T cell responses without prior *in vitro* manipulation has not been assessed.

To establish an MHCII-restricted *in vivo* model to test the ability of tumour-specific B cells to specifically activate tumour-specific CD4^+^ T cells, we utilized Hen Egg Lysozyme (HEL) specific SW_HEL_ B cells^3^ and Moth Cytochrome C residues 87–103 (MCC) T cell receptor (TCR) transgenic 5C.C7 CD4^+^ T cells in combination with a B16.F10 tumour line that expresses recombinant HELMCC antigen containing the relevant B and T cell epitopes.^4,5^ The ability of tumour-specific B cells to activate tumour-specific CD4^+^T cells independently of other APCs was determined. A key aspect of this model is that the 5C.C7 CD4^+^ T cell can recognize its cognate antigen MCC only when presented on the MHCII I-E allele. H-2^b^ C57BL/6 (termed B6) mice lack the ability to produce functional MHCII I-E molecules, whereas H-2^k^ B10.BR (termed BR) mice have a functional I-E gene. Through the specific interbreeding of B6 and BR mouse lines, wildtype expression levels of MHCII are maintained while antigen-presentation via MHCII I-E expression can be limited to either host APCs or adoptively transferred SW_HEL_ B cells. As hosts of adoptively transferred T and B cells, the model utilizes lymphopenic *Rag2*^*-/-*^ tumour-bearing mice that have been shown to support naïve T cell activation in multiple tumour models.^5,6^ In the B16.F10 HELMCC model used here, we have previously demonstrated that *Rag2*^*-/-*^ mice support potent IFN-γ-dependent CD4+ tumour responses capable of complete tumour clearance.^5^ The purpose of this study was to specifically ask if naïve and activated tumour specific B cells have the capacity to activate naïve tumour specific CD4 T cells *in vivo* without the confounders of other endogenous B cell or CD4 T cell populations being present. In addition, this model dissects this CD4 T cell: B cell interaction in the absence of CD8 T cells, allowing us to uniquely address the interactions of B cells and CD4 T cells in an MHC-restricted system.

## Methods

### Mice

All mice were bred and housed under SPF conditions in the Centenary Institute Animal Facility. SW_HEL_ mice were a gift from Robert Brink.^3^ These mice generate HyHEL10 B cell receptor (BCR)-expressing B cells and antibodies specific for the model antigen Hen Egg Lysozyme (HEL) and can switch to all antibody isotypes. SW_HEL_ mice were maintained on a C57BL/6 *Rag*2^−/−^ background^7^ and crossed with *Rag*2^−/−^mice on a B10.BR (H-2^k^) background when required. 5C.C7 TCR transgenic (tg) mice specific for Moth Cytochrome C residues 87–103 (MCC) plus I-E^k8^ have been described previously. 5C.C7 TCR tg mice were maintained on a *Rag*1^−/−^ B10.BR (H-2^k^) background and crossed with C57BL/6 *Rag*1^−/−^ mice for experimental use. Host and donor mice were also bred to express various combinations of CD45.1 and CD45.2 in order to unequivocally identify adoptively transferred cells.

### Cell lines and immunizations

The B16.F10 melanoma cell line was originally obtained from ATCC. B16.F10 cells were retrovirally transduced^9^ to express HELMCC, which consists of HEL protein with residues 64–76 replaced with residues 87–103 of MCC.^10^ To generate a membrane bound form of HELMCC, the connecting peptide, transmembrane and cytoplasmic domains of H-2K^b11^ were fused at the C-terminus. A stable high expressing clone designated B16.mHELMCC was used for all experiments.^5^ 1 × 10^6^ B16.mHELMCC cells were injected subcutaneously (s.c.) seven days prior to intravenous transfer of 5C.C7 CD4^+^ T cells and/or SW_HEL_ B cells, when large palpable tumours were present.

For *in* *vivo* activation of B cells, SW_HEL_ mice received a s.c. immunization with 5 × 10^6^ live B16.mHELMCC tumour cells followed by two intraperitoneal (i.p.) injections of anti-CD40 (FGK45, 25 μg/injection) on days 3 and 6. To generate effector/memory (eff/mem) 5C.C7 T cells, TCR tg mice were immunized s.c. in both flanks and the neck scruff with a total of 10 μg of MCC peptide 87–103 emulsified in Freund’s complete adjuvant (CFA) 3 weeks before cell harvest.

### Adoptive cell transfer, flow cytometry analysis and cell sorting

For adoptive transfer, SW_HEL_ B cells were isolated from spleens and TCR Tg 5C.C7 T cells from pooled lymph nodes. B cells were co-transferred with T cells at a 5:1 ratio (5×10^6^ B cells, 1 × 10^6^ T cells). Eff/mem 5C.C7 T cells were FACS sorted as CD4^+^TCR^+^CD44^hi^ CD62L^±^CD103^−^ using FACSAria II or Influx BD sorters. Samples were analyzed on LSR-II, Fortessa or FACSCanto BD flow cytometers. Antibodies were obtained from BD Pharmingen or eBioscience, or produced from B cell hybridomas in-house. The following monoclonal Abs were used to stain cells: anti-CD4(RM4–5), anti-CD11b(M1/70), anti-NK1.1(PK136), anti- CD45(30-F11), anti-MHCII(M5/114.15.2), anti-Ter119(TER 119) and anti-B220(RA3-6B2) obtained from BD Biosciences (Franklin Lakes, NJ, USA); anti-CD19(6D5), anti-CD45.2(104), anti- CD45.1(A20) and anti-Gr1(RB6-8C5) obtained from BioLegend (San Diego, CA, USA). All antibodies were directly conjugated. Non-specific binding to Fc receptors blocked using anti- CD16/32 purified in house from the 2.4G2-hybdridoma.

### Statistics

All tests were performed using GraphPad Prism Software. For nonparametric data, the Mann-Whitney test to compare ranks was used. For parametric data, unpaired Student *t* tests were used for comparisons between two populations and one-way ANOVA was used when comparing more than two groups. All data shown as mean ± SEM. (*, *P* < .05; **, *P* < .01; ***, *P* < .001; ****, *P* < .0001). There were no data point exclusions for each experiment.

## Results

**Figure 1.**
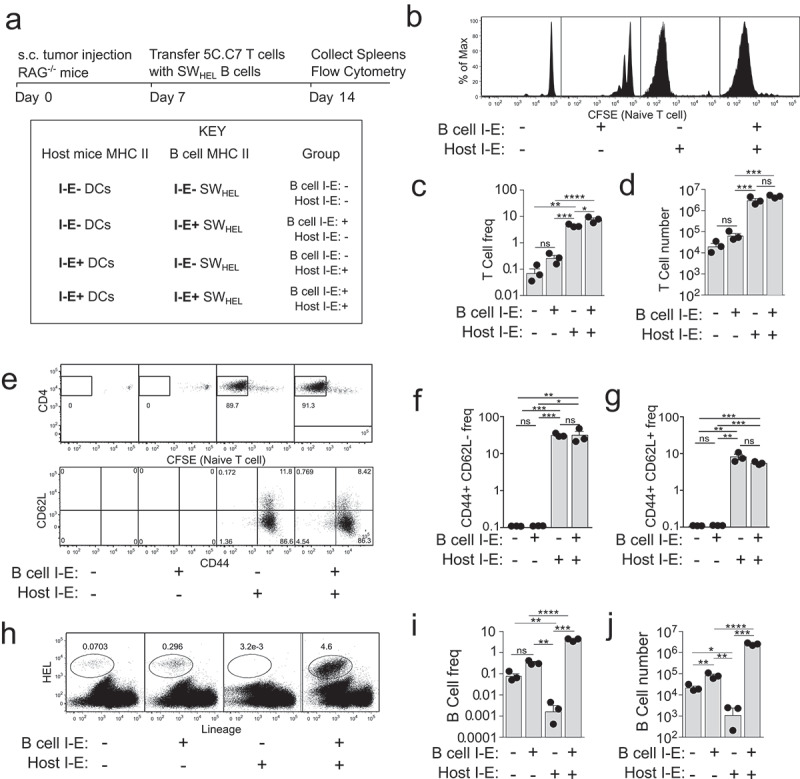
Naïve tumour-specific B cells are poor activators of naïve tumour-specific CD4^+^ T cells *in vivo* despite a lymphopenic environment.

To compare the ability of tumour-specific B cells and host myeloid APCs to present tumour antigen to CD4^+^ T cells, we developed a general experimental approach of adoptively co-transferring CFSE labeled 5C.C7 T cells with either I-E positive or I-E negative (termed I-E^+^ and I-E^−^, respectively) SW_HEL_ B cells, into I-E^+^ or I-E^−^ tumour bearing hosts (Figure 1a). A 5:1 B:T ratio was chosen to provide ample numbers of APCs for initial activation. Spleens and lymph nodes were harvested seven days after adoptive transfer for flow cytometric analysis (Figure 1a). The gating strategy to identify 5C.C7 T cells and SW_HEL_ B cells is shown in Supplementary Fig. S1A. When naïve T cells and naïve B cells were co-transferred, no T cell proliferation was observed in the spleen 7 days later, if I-E was absent from both host APCs and SW_HEL_ B cells (Figure 1b). Limited T cell proliferation was observed when SW_HEL_ B cells expressed I-E. Strong T cell proliferation occurred when host APCs expressed I-E (Figure 1b), with the vast majority of transferred T cells becoming CFSE-negative (having undergone > 7 cell divisions). Increased frequency (Figure 1c) and absolute number (Figure 1d) of T cells was seen. Divided T cells were predominantly CD62L^lo^ CD44^hi^ effector cells (Figure 1e,f). Spontaneous proliferation of adoptively transferred CD4^+^ T cells was not observed when tumour antigen was not present, even when both host APCs and SW_HEL_ B cells expressed I-E (Sup Figure 1c). T cell frequencies in tumour draining lymph nodes were similar to those obtained for spleens (Sup Figure 1d).

(a) Experimental set-up. I-E^+^ or I-E^−^ RAG^−/−^ host mice were s.c. injected in the flank with 1x10^6^ B16.mHELMCC tumour cells. Mice received I-E^+^ or I-E^−^ SW_HEL_ B cells and CFSE labelled 5C.C7 CD4^+^ T cells i.v. 7 days later. (b) Representative flow cytometry plots depicting CD4^+^ T cell proliferation (CFSE dilution). (c) Frequency and (d) absolute number of 5C.C7 CD4^+^ T cells in the spleen 7 days after transfer. (e) Representative plots of 5C.C7 CD4^+^ T cell CFSE dilution and expression of CD62L and CD44. Frequency of (f) effector memory (CD44+CD62L-) and (g) central memory (CD44+CD62L+) 5C.C7 CD4^+^ T cells in the spleen 7 days post transfer. (h) Representative plots of splenic HEL^+^ B cells 7 days after transfer. (i) Frequency and (j) absolute number of HEL^+^ B cells. *n*=3/group, 12 mice total for experiment. Representative of two independent experiments. ns = not significant, * = p < .05, ** = p < .01,*** = p < .001, **** = p < .0001.

B cell expansion in the spleen was the greatest when both host myeloid APCs and transferred SW_HEL_ B cells expressed I-E (Figure 1h). When only B cells expressed I-E, modest expansion was observed when compared to the I-E negative control. This was reflected in both frequency (Figure 1i) and absolute number of transferred B cells (Figure 1j). Similar B cell frequencies were observed in tumour draining lymph nodes (Sup Figure 1e). Interestingly, when host APCs were the only source of I-E, SW_HEL_ B cells were rapidly deleted. This was in the context of strong CD4^+^ T cell proliferation in response to host APCs. Given T cell and B cell frequencies were similar between the spleen and tumour draining lymph nodes, only spleens were analysed in subsequent experiments.

**Figure 2.**
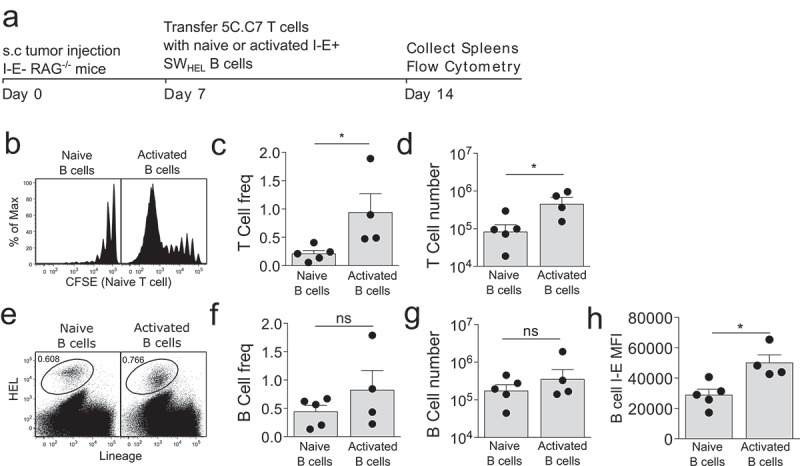
CD40 activated tumour-specific B cells have a limited capacity to activate naïve tumour-specific CD4 T cells.

*In vitro* stimulated B cells have been shown to activate CD4^+^ T cells in anti-tumour responses.^12^ We next examined if *in vivo* activated B cells could stimulate naive CD4^+^ T cell proliferation. Activated SW_HEL_ B cells were co-transferred with naïve CFSE labeled 5C.C7 CD4^+^ T cells into I-E- tumour bearing hosts (Figure 2a). Moderate T cell expansion was seen for activated compared to naïve B cells (Figure 2b), with some T cells undergoing > 7 rounds of division. This was reflected in both increased frequency (Figure 2c) and absolute number (Figure 2d). There was a trend toward an increase in activated compared to naïve SW_HEL_ B cell number but this did not reach statistical significance (Figure 2e–f). A significant increase in the MHCII I-E expression level on *in vivo* activated B cells (Figure 2h) may have contributed to the observed increase in T cell activating capacity. We have previously demonstrated these *in vivo* activated B cells express CD19, B220, CD23, HEL+ with subtle upregulation of MHC-II and CD95.^4,5^ Importantly, no proliferation is observed when naïve 5C.C7 CD4 T cells are transferred into I-E- anti-CD40 activated SWHEL transgenic tumour bearing mice (Figure S1f). These data suggest that *in vivo* activated tumour-specific B cells have a modest capacity to activate naïve CD4^+^ T cells.

(a) Experimental set-up. I-E^−^ RAG^−/−^ mice were s.c. injected in the flank with 1x10^6^ B16.mHELMCC tumour cells and after 7 days, naive 5C.C7 CD4^+^ T cells and activated SW_HEL_ B cells were co-transferred. To generate activated SW_HEL_ B cells, trangenic SW_HEL_ mice were injected10 days prior to transfer with s.c. B16.mHELMCC tumour cells in the flank followed by 2 i.p. injections of anti-CD40 agonistic antibody on days 3 and 6. (b) Representative flow cytometry plots depicting CD4^+^ T cell proliferation. (c) Frequency and (d) absolute number of 5C.C7 CD4^+^ T cells in the spleen 7 days after cell transfer. (e) Representative flow cytometric plots of HEL^+^ B cells in the spleens 7 days after cell transfer. (f) Frequency and (g), absolute number of HEL^+^ B cells. (h): Mean Fluorescence Intensity (MFI) of MHCII I-E expression on naïve and activated B cells 7 days after adoptive transfer. *n* = > 4 mice/group, 9 mice total for experiment. ns = not significant, * = p < .05.

**Figure 3.**
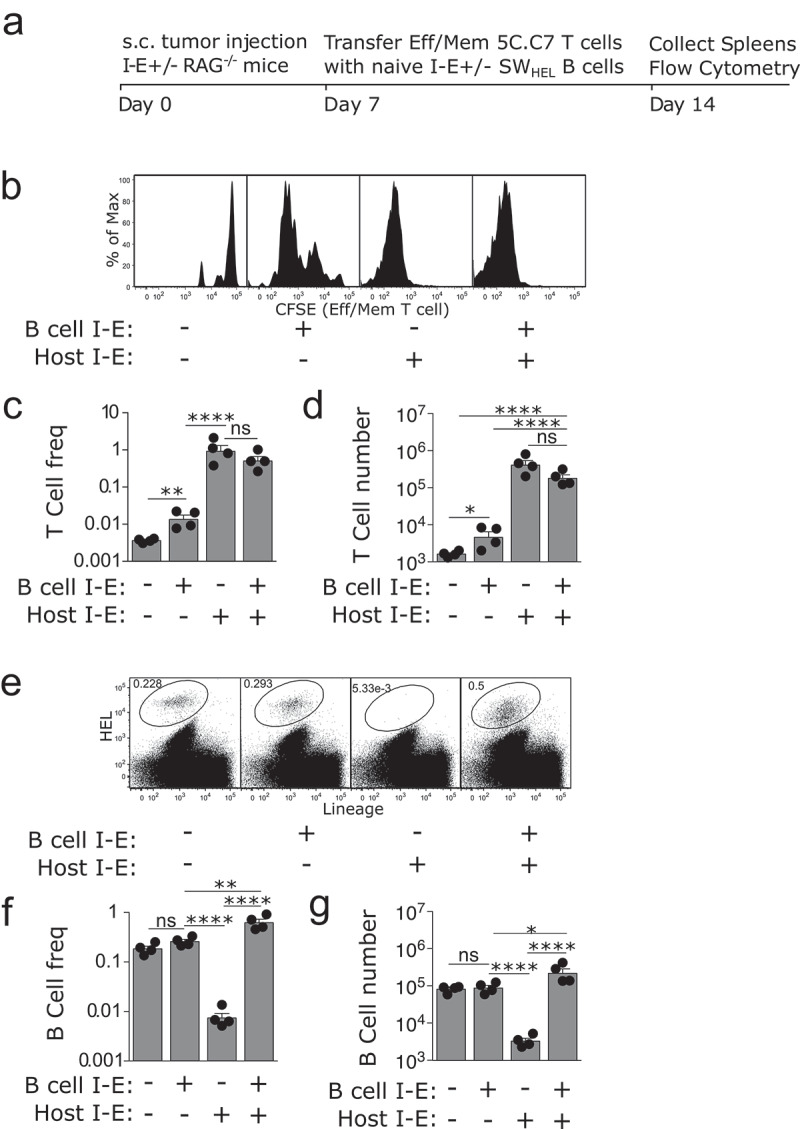
Naïve tumour-specific B cells have a limited capacity to activate effector/memory tumour-specific CD4 T cells.

The activation threshold of eff/mem T cells is known to be lower than that of naïve T cells,^13^ so we tested whether naïve B cells could activate eff/mem CD4^+^ T cells. Similar to the naïve setting, strong proliferation was induced when host myeloid APCs could present antigen, with all adoptively transferred T cells undergoing at least 8 cell divisions (Figure 3b). This expansion was reflected in both increased frequency (Figure 3c) and absolute number of T cells . When antigen presentation was limited to naïve B cells, eff/mem T cells proliferated more than naïve T cells (Figure 3b compared with Figure 1b). However B cells generated 100-fold fewer eff/mem T cells than host APCs.

(a) Experimental set-up as for Figure 1. except that 5C.C7 T cells were harvested from donor mice immunised with antigen emulsified in CFA 21 days prior. (b) Representative flow cytometry plots depicting CD4^+^ T cell proliferation. (c) Frequency and (d) absolute number of 5C.C7 CD4^+^ T cells in the spleen 7 days after cell transfer. (e) Representative flow cytometric plots of HEL^+^ B cells in the spleens 7 days after cell transfer. (f) Frequency and (g), absolute number of HEL^+^ B cells. *n* = 3 mice/group, 12 mice total for experiment. Representative of two independent experiments. ns = not significant, * = p < .05, ** = p < .01, **** = p < .0001ss

In the presence of eff/mem T cells, B cell expansion was the greatest when both host myeloid APCs and transferred SW_HEL_ B cells expressed MHC I-E (Figure 3e–g), although it was lower than in the presence of naïve T cells (Figure 2e–g). There was no significant difference in B cell expansion when B cells were the only APC expressing I-E, compared to the I-E negative control. Interestingly, deletion of I-E negative SW_HEL_ B cells was once again observed in the presence of strong host APC-induced T cell proliferation. Taken together, these data suggest that B cells have only a limited capacity to induce CD4^+^ T cell proliferation to tumour antigens *in vivo*.

## Discussion

The dominant MHCII-dependent activators of both naïve and effector memory CD4^+^ T cells in this model were host myeloid APCs, with B cells playing only a minor role, even after prior *in vivo* activation of the T or B cells. The small degree of activation experienced by CD4^+^ T cells responding to B cell-presented antigen in our experiments is unlikely to fully support an effective anti-tumour response, even in a highly lymphopenic *Rag2*^−/−^ environment. In contrast, host APC-dependent activation drives a potent CD4^+^-dependent anti-tumour response in this mouse model.^5^

Similar results have been observed in a mouse model of experimental autoimmune encephalomyelitis (EAE) in which Archambault et al. used a cre-mediated conditional approach to limit antigen presentation to CD19^+^ cells.^14^ They observed only limited CD4^+^ T cell activation in initial and secondary responses. These experiments were conducted in wild type mice with wild type B cells. Importantly, when presentation was limited to B cells, T cell activation was insufficient to induce EAE disease.

These data do not negate the involvement of B cells in optimal antigen presentation in more complex anti-tumour immune responses involving both CD4^+^ and CD8^+^ T cells. There has been growing interest in the role of B cells in anti-tumour immunity and a number of possible roles have been considered [Reviewed in^15^]. B cell-based vaccines may influence a number of established MHCII dependent and independent functions, including antigen presentation to both CD4^+^ and CD8 T^+^ cells, antibody production and potentially suppression via regulatory B cell populations. In contrast, although DCs have generally been considered to be the more relevant APC for T cell priming in a wide range of contexts, tumour lysate vaccination and DC-based adoptive cellular therapies have shown only limited success in treating cancer [Reviewed in^16^]. In addition, DC-based therapy faces major practical constraints, whereas B cells can be easily purified from the peripheral blood of patients.

*In vitro* T cell proliferation and IFNγ production has been observed in response to polyclonal B cells purified from PBMCs and cultured with CD40L and tumour cell lysates.^17^ Similarly, CD40-activated B cells loaded with myeloma lysates were capable of activating myeloma antigen-specific T cells *in* *vitro*.^18^ These results collectively demonstrate that polyclonal human B cells stimulated *in vitro* can contribute to T cell mediated anti-tumour immune responses. The ability to identify human B cells with tumour reactive specificities *in vitro* may also generate new therapeutic approaches.^18^

In murine models, several lines of evidence support a role for B cells in anti-tumour responses. In the B16.F10 mouse melanoma model, *in vivo* depletion of murine B cells with an anti-CD20 monoclonal antibody attenuated the antigen-specific responses of CD4^+^ and CD8^+^ T cells.^19^ Antibodies coupled to tumour proteins have been shown to induce CD8^+^ T cell activation via cross presentation of antibody complexes by mouse DCs.^20^ In addition, a cancer vaccine based on coupling tumour antigens such as her-2/neu to an scFv anti-CD19 mAb has been shown to reduce tumour growth.^21^

Our data suggest that therapies aimed at generating CD4^+^ dependent anti-tumour T cell responses should not target B cells alone. They also underline the importance of T cell:B cell contact for B cell survival during activation. B cell deletion was apparent when B cells lacked MHCII I-E but host APCs did not (Figures 1h–j and 3e-g). The deleted SW_HEL_ B cells were still capable of binding the HEL antigen but were unable to make MHCII-specific interactions with proliferating T cells responding to host APCs. It is possible that activation with tumour-derived antigen and absence of T cell help may be inducing anergy leading to deletion of the SW_HEL_ B cells. Landmark studies of the HEL transgenic B cell system demonstrated that in contexts where HEL was expressed as a self-antigen, B cells underwent rapid deletion within 1 week, similar to what was observed in these experiments.^22^ Furthermore, T cells may play a role in deletion of anergic B cells through CD40- and Fas-ligands interactions.^23,24^ This phenomenon is currently under investigation. We appreciate that the use of high affinity transgenic CD4 T cell and B cells in combination with lymphopenic RAG-/- mice and a transplantable tumour line is an artificial system and does not recapitulate the complexities of the adaptive immune systems interaction with cancer. However despite this, it is striking how poor B cells were at activating the tumour-specific CD4 T cells in one of the most lymphopenic systems available to study in mice. This reiterates the importance of DCs being the most important APC in the activation of naïve CD4 T cells *in vivo*. Of note, maximum T cell and B cell expansion in the effector/memory experiments described in Figure 3 were an order of magnitude lower compared to the experiments described in Figure 1. This may reflect partial homing of effector T cells directly to the tumour tissue rather than the spleen, but may also reflect a blunted ability to proliferate compared to naïve CD4 T cells, which is consistent with previous comparisons of naïve vs effector T cell proliferation.^25^

In summary, while published studies indicate that B cells may have important role in the cooperative immune response against tumours, our use of MHC-restriction to isolate the *in vivo* effects of tumour-specific B cells on tumour-specific CD4^+^ T cells indicate that primary cognate interactions between CD4^+^ T cells and B cells are unlikely to serve as a major mechanism of CD4^+^ T cell activation.

## Supplementary Material

Supplemental Material

## Data Availability

Raw data were generated at the Centenary Institute. Derived data supporting the findings of this study are available from the corresponding author BF on request.
