# Supplementary material for: MHCII restriction demonstrates B cells have very limited capacity to activate tumour-specific CD4+ T cells in vivo
Source: Oncoimmunology. 2023 Dec 10;13(1):2290799. doi: 10.1080/2162402X.2023.2290799 (PMC10730170; doi:10.1080/2162402X.2023.2290799)
Supplement: Supplemental Material [file KONI_A_2290799_SM7758.docx]

**
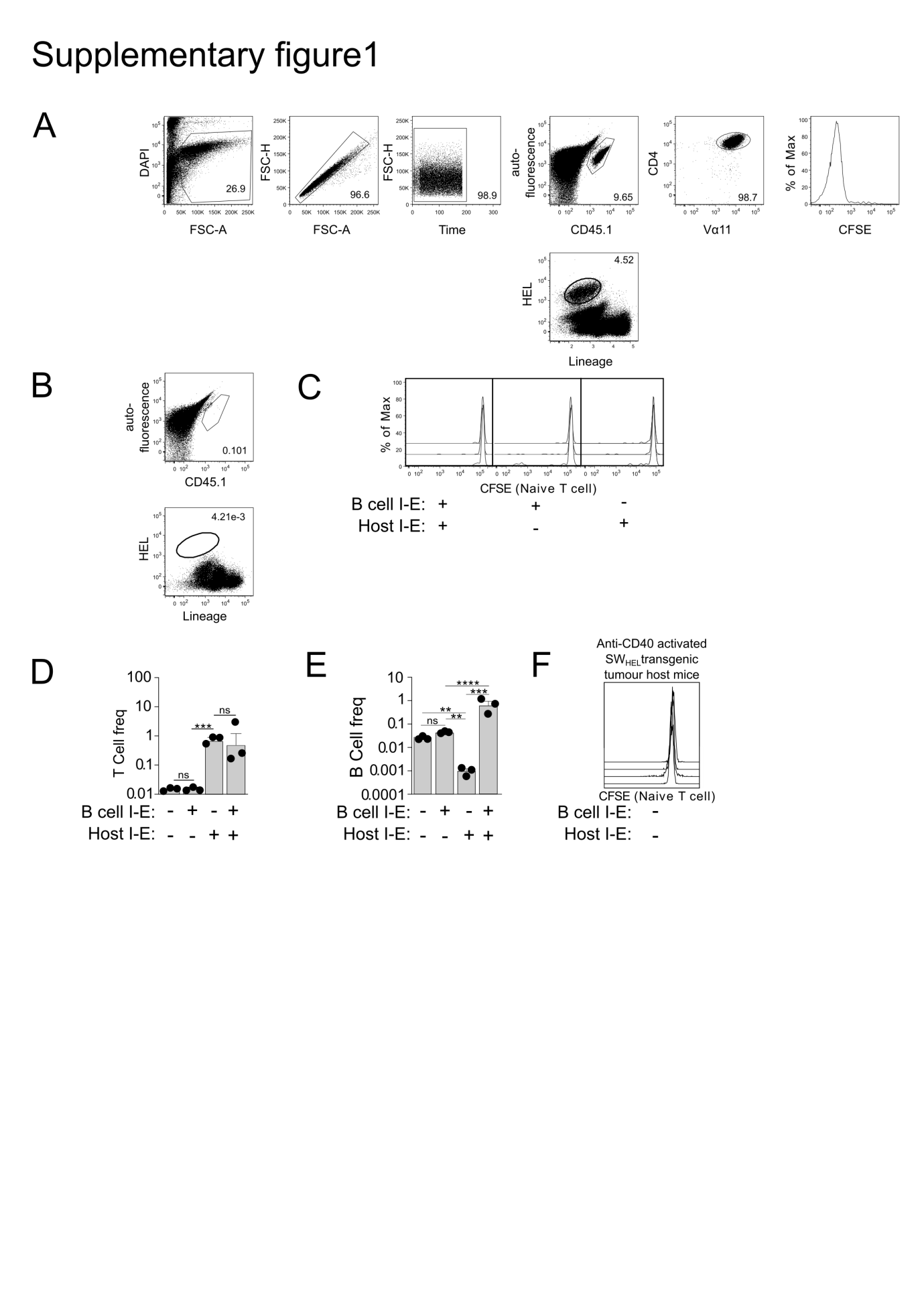
**

**Supplementary Figure 1: Flow cytometric gating strategy and no tumour cell proliferation controls**

A: Representative flow cytometric plots depicting 5C.C7 CD4^+^ T cell and SW_HEL_ B cell gating strategy. T cells were gated DAPI^-^, singlets^+^, time^+^, autofluorescence^-^ CD45.1^+^, CD4^+^, Vα11^+^. B cells were gated DAPI^-^, singlets^+^, time^+^, lineage^-^, HEL^+^. B: Control - No T or B cell transfer into RAG^-/-^ host mice. Lineage cocktail, for gating HEL antigen-specific B cells of interest, included antibodies against Ter119, CD8, NK1.1, GR1and CD11b. C: Response of CFSE labelled 5C.C7 T cells in hosts with no tumour. D, frequency of 5C.C7 CD4^+^ T cells and E: HEL^+^ B cells in the tumour draining inguinal, axillary and brachial lymph nodes 7 days after cell transfer. F: Representative flow cytometry plot of 5C.C7 T cell CFSE dilution 7 days post transfer into anti-CD40 activated SW^HEL^ transgenic tumour bearing mice (n=4). SW_HEL_ mice received a s.c. immunisation with 5x10^6^ live B16.mHELMCC tumour cells followed by two intraperitoneal (i.p.) injections of anti-CD40 (FGK45, 25μg/injection) on days 3 and 6. ns=not significant, **=p<0.01, ***=p<0.001, ****=p<0.0001.
